# A study to further develop and refine carpal tunnel syndrome (CTS) nerve conduction grading tool

**DOI:** 10.1186/s12891-019-2928-y

**Published:** 2019-12-03

**Authors:** Salim Hirani

**Affiliations:** Neurophysiology Department, Ysbyty Gwynedd Hospital, Bangor, North Wales LL57 2PW UK

**Keywords:** Grading tools for carpal tunnel syndrome, CTS Gradings, Neurophysiological CTS grading

## Abstract

**Background:**

The severity of carpal tunnel syndrome (CTS) may be categorised in a number of ways utilising one of a range of presently available grading tools. The grading systems proposed by Bland and Padua are the most commonly used, however, both have limitations, which are discussed in detail in this paper.

The aim of this research is to establish, using the best available evidence, a clinically appropriate revision of the current CTS nerve conduction grading tool, and to compare with existing grading tools used in UK Neurophysiology clinics. The revised scale is designed from a clinical physiologist perspective and based on the numerical values of nerve conduction findings.

The proposed revised grading system is based on more nuanced, descriptive categories, ranging from Normal to Early, Mild Sensory, Mild Sensory Motor, Moderate Sensory, Moderate Sensory Motor, Severe Sensory Motor, Extremely Severe Sensory Motor, and Complete absence.

**Method:**

A total of 1123 patients (2246 hands) were included in this study, with the aim of evaluating the revised grading system. Data was collected based on the extensive and detailed grading systems previously described by Bland and Padua. All data was recorded numerically to ensure methodological reliability.

**Result:**

Of the 2246 patients’ hands tested, the nerve conduction was graded as normal in 968 hands; nerve conduction showed early changes in 271 hands; mild sensory changes in 215 hands, mild changes in both motor and sensory response in 51 hands; moderate sensory changes in 134 hands; moderate sensory and motor changes in 356 hands; severe changes in motor and sensory responses in 204 hands; extremely severe sensory and motor changes in 33 hands and complete absence of response in 14 hands.

**Conclusion:**

The revised grading tool could offer a more numerical grading to the Clinical Physiologist and could help the surgeon to ascertain the level of severity in order to decide on either a conservative or surgical approach to treatment if they decide to use the proposed grading which could support them to defend their decision in cases of litigation.

## Background

The pathology of Carpal Tunnel Syndrome (CTS) is described as “*A Neuropathy caused by entrapment of the median nerve at the level of the carpal tunnel*” [1, 2]. Nerve Conduction Studies (NCS) are one of the basic tools used to support clinical diagnosis. NCS are objective tests that assess the physiological status of the median nerve across the carpal tunnel [3].

### Reason for grading carpal tunnel syndrome

The Grading tool is used for the diagnostic assessment of CTS in conjunction with the patient’s clinical history and symptoms in order to diagnose the degree of severity of CTS [2]. The revised grading tool using a physiological basis offers a more precise numerical grading, which is both objective and repeatable. This could not only help the Clinical Physiologist to grade there result according to the propose grading scale but probably it also support the surgeon to ascertain the level of severity and could help to decide on either a conservative or surgical approach to treatment.

There are several primary grading tests mentioned in the different literature, associated with Phalen’s, Tinel’s and Durkan’s signs which are subjective and are based on patient clinical response. Other tests like Ultrasound, NCS and EMG needle examination are objective tests that have been used for CTS grading which are reliable, evidence-based and objective, not dependent on patient clinical response [1].

However, to ascertain the severity level of CTS, specific neurophysiological grading is required [4]. There are several grading scales for investigations specifically related to CTS; [Campbell [5], Padua [4], Bland [6], Giannini [3], Carvalho [7], Ajeena [1], Jeong [8] and Jerosh-Herold [9]]. Most of the studies show grading in subjectivity. Some lack a neurophysiological focus in objectivity [7] during the collection of the data. Some researches only use either Sensory Nerve Conduction Study (NCS) or Motor NCS to differentiate the severity of CTS grading. Not all researchers have used sensitive techniques to diagnose early CTS or in severe cases, Lumbrical responses to differentiate its severity from complete absence, which therefore cannot be diagnosed as CTS with complete certainty.

It appears that whilst there is an accepted dominance of both the Bland [6] and Padua [4] grading systems, there are also clear limitations which are discussed comprehensively in this paper.

In the UK, the Bland [6] grading is largely followed due to its depth of detail. In 2014 the Association of Neurophysiological Science (ANS), in collaboration with the British Society for Clinical Neurophysiology (BSCN) published guidelines outlining the accepted grading of CTS in the United Kingdom, which follows the Bland [6] grading system. The reason given was that it focuses on the clinical physiologist specialism, as well as its element of flexibility.

The aim of this research was to establish evidence-based revision of the current CTS nerve conduction Grading Tool used in the UK and to evaluate its effectiveness - in terms of acceptability and usability for Clinical Physiologist as well as a tool for intervention prediction for Surgeon. This could support the Surgeon to ascertain the level of severity and decide on a conservative or surgical approach to treatment. Although surgeons must take their own decision for the treatment of CTS, if they want to consider the treatment on the basis of the proposed Nerve conduction study grading, this will probably allow to defend their decisions in the Magistrate Court. A numerical value is given to each of the grade bandings to enable objective reporting and comparision [4].

No clinical assessment was conducted during the Neurophysiological test so as to secure the biasness from patient’s condition.

## Method

Ethical approval for the research project was obtained from the Heath Research Authority National Research Ethics Service London – Queen Square Research Ethics Committee (Reference 17/LO/0750).

Neurophysiological data was collected based on the extensive and complete description of previous study designs by Padua [4] and Bland [6] and which is understood to be followed by most of the clinical laboratories in the United Kingdom. In addition, Second Lumbrical-Interosseous Latency was also recorded to distinguish between ‘very severe’ and ‘complete absent’ response grading of CTS [10].

The Association of Neurophysiological Scientists (ANS) (2014) guidelines and the minimum standards for the practice of Clinical Neurophysiology in the United Kingdom were followed. Few new grading was introduced during collection of the data to cover full range of grading.

The test was performed by a qualified Clinical Physiologist (Neurophysiology) using Keypoint 9033A07 (Skovlunde, Denmark) machine, on the bases of departmental protocol (Peripheral protocol1, 2015). A quantitative method was used for collecting data [11], to ensure accuracy and to avoid bias. The sample size of patients in the study was use for all those tested for NCS over a period of one calendar year (2017), across the population of North Wales. The data was collected from patients with an age range above 18 years, who were referred to the Neurophysiology department from the Orthopaedics and Neurology departments within the local Health Board, as well as General Practices (GPs) in North Wales. No individual patient was recruited in this research. The inclusion criteria were considered only on the basis of the referral diagnosis. No clinical assessment was conducted prior to the study in the department. Referral of CTS was considered based on paraesthesia, pain, swelling in median distribution area or digits I-V, worsened by sleep. The test was carried out by testing both hands (symptomatic and asymptomatic) to fulfil department protocol.

Data was analysed on certain widely accepted assumptions of sensory amplitude and CV and distal motor latency (DML), amplitude and CV [2, 6]. To introduce the terms “mild”, “moderate” and “severe”, a numerical value was used which could be accepted widely, and which can be used to compare with others studies [4].

The procedure started by carrying out the sensory testing, by placing the stimulating ring electrodes on digit III (which is more sensitive then digit II [6]) and the recording electrode on the surface of the median nerve on the wrist. The orthodromic technique was used for the sensory and motor NCS test, through the median and ulnar nerves. A maximal current was applied to record the full response of the nerve, at the digits II-IV for median sensory and digit V for ulnar sensory recording. A maximal current was applied to stimulate median nerve pathways at the wrist and at the elbow for motor recording from abductor pollicis brevis (APB) [11] and ulnar nerve pathways from First dorsal interosseous (FDI). Digit II was stimulated only when either the response from digit III was less than 3 μV or absent; digit IV was stimulated only when the response from digit III showed conduction velocity between 45 and 50 m/sec. Amplitude was recorded from peak to peak for sensory responses, and base to peak for motor responses. If responses were not recordable from median sensory digit II, III and motor from APB muscles, then motor responses were elicited by placing recording electrodes on 2nd lumbricals by stimulating median and ulnar nerves at the wrist [7, 10–12].

All patient data was collected by fulfilling the criteria mentioned in above paragraph depending on the severity. The reason for using the new criteria is to describe the full range of severity which was not fully covered by other research mentioned earlier in this paper. Criteria was mentioned in above paragraph are intended to be more reliable in terms of grading for Clinical Physiologist and probably will allow support to the Surgeon in terms of patient treatment decisions.

The grades are:

Normal (Grade 0): where sensory conduction velocity (SCV) is above 50 m/s and amplitude ≥5 μV with DML ≤4.2 ms, amplitude ≥5 mV and motor conduction velocity (MCV) ≥50 m/s.

Early (Grade 1): where SCV is between 45 and 50 m/s from digit III and double peak latency in digit IV is > 0.5 ms with DML ≤4.2 ms and normal sensory and motor amplitude > 5 (sensory in μV and motor in mV).

Mild Sensory (Grade 2): where SCV is between 40 and 44.9 m/s from digits III with normal sensory amplitude and motor values mentioned in Grade 0.

Mild Sensory-Motor (Grade 3): where SCV is between 40 and 44.9 m/s from digits III with normal sensory amplitude mentioned in Grade 0, DML ≥4.2 ms with normal motor amplitude and CV.

Moderate Sensory (Grade 4): where SCV is less than 40 m/s from digits III with normal sensory amplitude and normal motor values mentioned in Grade 0.

Moderate Sensory-Motor (Grade 5): where SCV is less than 40 m/s from digits III with normal sensory amplitude, DML ≥4.2 ms with normal motor amplitude and CV.

Severe Sensory-Motor (Grade 6): where sensory potentials from digits III and digit II are absent or < 3 μV in both digits III and II with SCV < 30 m/s, DML ≥4.2 ms, MCV is either slow or normal.

Extremely Severe Sensory-Motor (Grade 7): where sensory and motor potentials are absent and response recordable only from 2nd lumbricals, where median lumbricals are prolonged compared and low amplitude to ulnar lumbricals.

Complete (Grade 8): where both sensory and motor potentials are absent and responses are not recordable from median 2nd lumbricals but recordable from ulnar 2nd lumbricals. (Please refer to a Comparison of the Bland [6] grading with the proposed revised grading is given at the end of this study for more understanding).

## Results

The data was collected for a period of 1 year (2017). Initially a total of 1132 patients were included in this study. During data collection, two referrals were not included, because the patients declined to participate in all study procedures; and seven participants’ data sets were excluded from the analysis because the departmental protocol was breached. Therefore 1123 patients (2246 hands) were included in the final data collection.

**Fig. 1:**
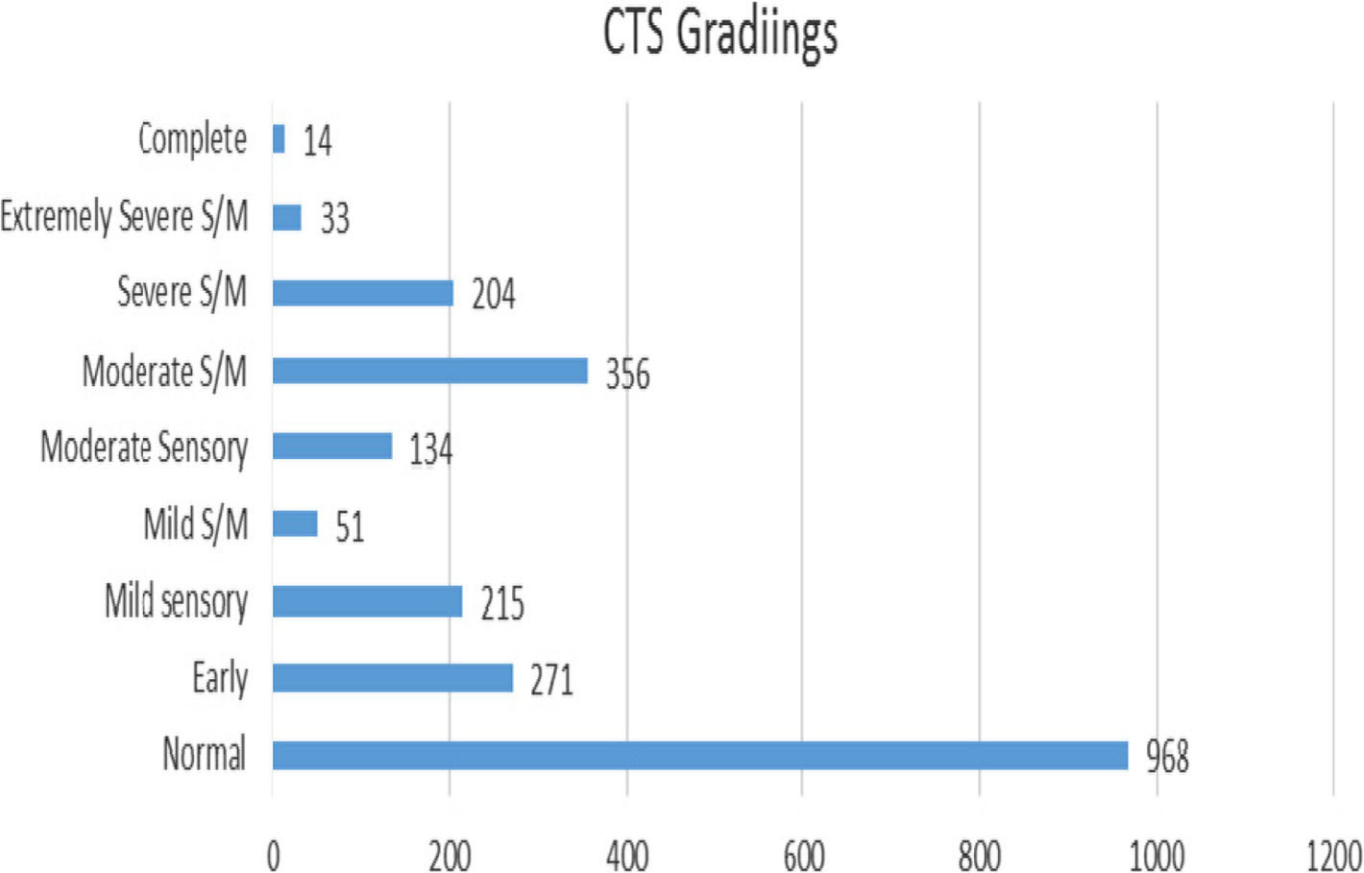
Result

Of the 1123 patients, 687 were female and 436 were male. The age range was 19 to 98 years, median age 56 years. The numbers of hands in each grade of severity are shown in Fig. 1 and Table 1.

**Table 1:** Result

Normal	968
Early	271
Mild sensory	215
Mild S/M	51
Moderate Sensory	134
Moderate S/M	356
Severe S/M	204
Extremely Severe S/M	33
Complete	14
Total Hands	2246

## Discussion

The Bland [6] grading system, which was collected for Canterbury region, enables the neurophysiologist to differentiate between the levels of severity for the Clinical Physiologist point of view [4]. However, due to the limited numerical grading, it is felt that the Bland [6] grading does not enable the level of severity to be objectively and fully ascertained as possible to cover all level of grading of CTS.

Bland [6] recorded that prolongation of the motor terminal latency to APB is not significant in mild cases which the author partially agrees with, as it appears insignificant in the graph (Please refer to the graph) compared to mild sensory CTS. It was noted that neither Bland [6] or Padua [4] separate between mild sensory and mild sensory-motor in their Grading. This clearly indicates that there is a need for some revision and a separate grading in mild CTS. However, when compared to moderate sensory- motor CTS with moderate sensory CTS, moderate sensory-motor CTS has significantly higher patient numbers than the moderate sensory. This indicates that there is room for revision and a separate grading in moderate CTS. (Please refer to percentage table).

In theory, the higher the grade, the worse nerve dysfunction^4.^ The analysis of the data, however, in this study appears to show mixed levels of severity. A majority of CTS studies in this investigation appear normal, due to the fact that non-symptomatic hands were also recorded to fulfil department protocol. Although normal (Grade 0) has much higher numbers compared to other grades. This does not detract from the fact that levels of severity for CTS were found. In this study, comparison of the grading scales shows that there are big differences in the mild sensory-motor groups between Bland’s [6] and this proposed grading. 11% in Bland’s [6] mild sensory-motor category with 10% mild sensory and only 2% mild mixed sensory motor in propose grading, which might suggest that the separation is marked, although the separation has value in demonstrating the effects in the motor fascicles and this may have an impact in choosing treatment option. However, at the moderate degree of severity, there is a notable difference with 16% in Bland’s [6] moderate category and 6% moderate sensory and 16% moderate mixed sensory motor, suggestive that these are 2 groups worth separating.

The Table 2 summarises and compares the variance in Bland [6] and Padua [4] Gradings with the revised grading system.

**Table 2:** Percentage comparison with grading of Padua, Bland and Hirani

Padua (%)		Bland (%)	Hirani (%)
	Normal = 18 (3)	Normal = 3269 (38)	Normal =968 (43)
	Minimal = 123 (21)	Very mild = 684 (8)	Early =271 (12)
	Mild = 145 (24)	Mild sensory-motor = 944 (11)	Mild sensory = 215 (10)
			Mild S/M = 51 (2)
	Moderate = 217 (36)	Moderately Severe =1359 (16)	Moderate sensory = 134 (6)
			Moderate S/M = 356 (16)
	Severe = 81 (14)	Severe = 568 (7)	Severe S/M = 204 (9)
	Extremely severe = 16 (3)	Very severe = 930 (11)	Extremely Severe S/M = 33 (1)
		Extremely Severe = 387 (5)	Complete = 14 (1)
Total Hands	600	8501	2246

Padua [4] relates the outcome of grading with surgical decompression compared to Bland’s [6] grading which is based purely on neurophysiological concepts. Bland’s [6] grading scale was based on a very large population. However, in a ‘severe’ grading, the values were not clear. Bland [6] has given the amplitude for the motor response, but has not taken into account the DML and CV, and for ‘extremely severe’ grading the values was not clear either. At ‘extremely severe’ grading, although the amplitude in the motor CMAP has been taken into account, no account has been taken of distal latency and CV [6].

Comparing, the Bland [6] grading system of CTS with the grading suggested in this paper, it seems that Bland [6] Grade 0 and 1 are comparable with the propose grading. The author would suggest that the Bland [6] grading for Grade 2 needs to become more elaborate by dividing them into two separate groups, i.e. mild sensory and mild sensory-motor, as more patient’s data shows just mild sensory changes compared with mild sensory and motor together. In the revised grading the sensory involvement is graded as Grade 2 and the sensory and motor where both functions are involved is graded as Grade 3.

Bland [6] covers a moderate degree of severity in grade 3, which again warrants further elaboration to make the gradings more objective and more descriptive. In propose revised system grade 4 covers sensory involvement and grade 5 covers both sensory and motor involvement together.

Bland’s [6] Grade 4 which is a severe CTS compares favourably with Grade 6 of the revised grading system.

Bland [6] only describes a prolonged DML in his Grade 5 as ‘very severe’ CTS which the author believes do not fulfil all the criteria to separate from his Grade 6. Bland [6] appears not to have taken any account changes in sensory potentials or motor conduction velocity values. The revised grading system has graded ‘Very Severe’ CTS where both sensory and motor responses are absent, and responses were only recordable from the 2nd lumbrical with prolonged median distal latency compared to ulnar lumbricals as Grade 7.

The Grade 6 in Bland [6] again has the potential to create confusion as it refers to a low amplitude motor potential. It appears not to have taken into account the DML in Grade 6. CV was also not included in the Grade 6 grouping in the Bland [6] grading. The author has considered the latency and amplitude with CV and graded as complete median nerve dysfunctions where both median motor and sensory as well as median 2nd lumbrical responses are absent, and the only response appears in the ulnar 2nd lumbrical. This grade appears as grade 8 in the revised grading system.

Table 3 summarises and compares the Bland [6] Gradings with the revised grading system.

**Table 3:** Grading comparison of Bland with propose grading

Grading	Bland [6]	Modified grading by Hirani
Grade1	Inching, palm/wrist median/ulnar comparison, ring finger double peak	Early: SCV = 45–50 m/s interpeak potentials in digit IV > 0.5 ms, DML < 4.2 ms. Amplitude of sensory ≥5 μV and motor potentials ≥5 mV
Grade2	Mild: sensory conduction velocity(SCV) < 40 m/s distal motor latency (DML) < 4.5 ms	Mild sensory: SCV = 40–44 m/s with normal sensory amplitude (NSA), DML, motor nerve action potentials (MNAP) and sensory conduction velocity (SCV) & motor conduction velocity (MCV)
Grade3	Moderately severe: DML > 4.5 ms and < 6.5 ms with sensory nerve action potentials (SNAP) preserved	Mild sensory motor: SCV = 40-44 m/s with NSA, DML > 4.2 ms with normal motor amplitude (NMA) and normal SCV and MCV
Grade4	Severe: DML > 4.5 ms and < 6.5 ms with absent SNAP	Moderate sensory: SCV < 40 m/s with NSA, normal DML, NMA, SCV and MCV
Grade5	Very severe: DML > 6.5 ms.	Moderate sensory motor: SCV < 40 m/s with NSA, DML > 4.2 ms, MNA and SCV and MCV
Grade6	Extremely Severe: motor nerve action potentials (MNAP) < 0.2 mV,	Severe Sensory motor: Absent or < 3 μV SNAP with SCV < 30 m/s with DML > 4.2 ms with either slow or normal MCV and or NMA
Grade7		Extremely severe: SNAP and MNAP = absent, but recordable from both median and ulnar 2nd lumbricals with prolonged median 2nd lumbricals response as compare to ulnar lumbricals
Grade8		Complete: SNAP and MNAP = absent and absent from median 2nd lumbricals and present from ulnar 2nd lumbricals

## Conclusion

The grading system devised by Bland [6] and used to grade the levels of severity of CTS over the last 17 years within the UK has certain limitations, and the author believes system needs modification in order to accommodate current practice. The revised grading system for CTS is based on a review of a current and past literature.

Bland [6] and Padua [4] both limited the DML and CV in motor study and amplitude potentials and CV for sensory study. Author follow the same rule and precedes the study with given cut off values to grade them accordingly. Most of the Clinical laboratories in UK use the above criteria of cut off values for sensory and motor study to create their own normative values. Presently, there is no standard of CTS grading followed throughout the UK due to their limitations, the propose grading scale, preferably was felt to be an acceptable and useable tool for Clinical Physiologist and could use for intervention allocation.

The revised grading tool using a physiological basis offers a more precise numerical grading, which is both objective and repeatable. This could not only help the Clinical Physiologist to grade there result according to the propose grading scale but probably it also support the surgeon to ascertain the level of severity and could help to decide on either a conservative or surgical approach to treatment. Please note that this research was made to amend the grading for Clinical Physiologist. Although surgeons have to take their own decision for the treatment of CTS, but if they want to consider the treatment on the basis of the proposed nerve conduction study grading, this will probably aid defence of their decisions for the court. This is advisable (but not necessary to follow) that Surgeons could consider proposed Grade 1–2 for physiotherapy treatment, Grade 3–4 for conservative or intervention of steroid treatment and Grade 5–7 for surgical intervention where the chances of full recovery. Surgeon could decide for surgical intervention of Grade 8 cases, whether it would be beneficial or not in keeping with the patient’s age and other medical history.

Future studies looking at prognosis may be helpful in looking at the outcomes from different interventions for those with different gradings of severity and to look at the implications of motor involvement compared with just sensory fascicle involvement. Collections of data are under process for post surgery CTS outcome which will publish later after approval from BCUHB research committee.

## Data Availability

The datasets analyzed during the current study are not publicly available as they are held within patient records but are available from the corresponding author on request.

## Abbreviations

ANS: Association of Neurophysiological Scientists
APB: Abductor polices braves
BCUHB: Betsi Cadwaladr University Health Board
BSCN: British Society for Clinical Neurophysiology
CTS: Carpal tunnel syndrome
CV: Conduction velocity
DML: Distal Motor Latency
GPs: General Practices
MCV: Motor conduction velocity
MNAP: Motor nerve action potentials
NCS: Nerve Conduction Studies
NMA: Normal motor amplitude
NSA: Normal sensory amplitude
SCV: Sensory conduction velocity
SNAP: Sensory nerve action potentials
